# Mutations and intron polymorphisms in voltage-gated sodium channel genes of different geographic populations of *Culex pipiens pallens*/*Culex pipiens quinquefasciatus* in China

**DOI:** 10.1186/s40249-024-01197-1

**Published:** 2024-04-15

**Authors:** Wenyu Li, Delong Ma, Qunzheng Mu, Xinxin Zhou, Dongdong Hua, Chunchun Zhao, Qiyong Liu, Jun Wang, Fengxia Meng

**Affiliations:** 1 State Key Laboratory of Infectious Disease Prevention and Control, Department of Vector Biology and Control, National Institute for Communicable Disease Control and Prevention, Chinese Center for Disease Control and Prevention, WHO Collaborating Centre for Vector Surveillance and Management, Beijing, 102206 People’s Republic of China; 2 Ningxia Center for Disease Prevention and Control, YinChuan, 750004 NingXia People’s Republic of China; 3 Jinan Shizhong Center for Disease Control and Prevention, Jinan, 250000 Shandong China; 4 Vanke School of Public Health, Tsinghua University, Beijing, People’s Republic of China; 5 Weifang No. 2 People’s Hospital, Weifang, 261000 Shandong People’s Republic of China; 6 Beijing Daxing Center for Disease Control and Prevention, Beijing, 102600 People’s Republic of China; 7 Jinan Second Maternal and Child Health Hospital, Jinan, 250000 Shandong China

**Keywords:** *Culex pipiens pallens*, *Culex pipiens quinquefasciatus*, *vgsc* gene, Mutation, Frequency, Intron, Haplotype, China

## Abstract

**Background:**

*Culex pipiens pallens* and *Culex pipiens quinquefasciatus* are the dominant species of *Culex* mosquitoes in China and important disease vectors. Long-term use of insecticides can cause mutations in the voltage-gated sodium channel (*vgsc*) gene of mosquitoes, but little is known about the current status and evolutionary origins of *vgsc* gene in different geographic populations. Therefore, this study aimed to determine the current status of *vgsc* genes in *Cx. p. pallens* and *Cx. p. quinquefasciatus* in China and to investigate the evolutionary inheritance of neighboring downstream introns of the *vgsc* gene to determine the impact of insecticides on long-term evolution.

**Methods:**

Sampling was conducted from July to September 2021 in representative habitats of 22 provincial-level administrative divisions in China. Genomic DNA was extracted from 1308 mosquitoes, the IIS6 fragment of the *vgsc* gene on the nerve cell membrane was amplified using polymerase chain reaction, and the sequence was used to evaluate allele frequency and knockdown resistance (*kdr*) frequency. MEGA 11 was used to construct neighbor-joining (NJ) tree. PopART was used to build a TCS network.

**Results:**

There were 6 alleles and 6 genotypes at the L1014 locus, which included the wild-type alleles TTA/L and CTA/L and the mutant alleles TTT/F, TTC/F, TCT/S and TCA/S. The geographic populations with a *kdr* frequency less than 20.00% were mainly concentrated in the regions north of 38° N, and the geographic populations with a *kdr* frequency greater than 80.00% were concentrated in the regions south of 30° N. *kdr* frequency increased with decreasing latitude. And within the same latitude, the frequency of *kdr* in large cities is relatively high. Mutations were correlated with the number of introns. The mutant allele TCA/S has only one intron, the mutant allele TTT/F has three introns, and the wild-type allele TTA/L has 17 introns.

**Conclusions:**

*Cx. p. pallens* and *Cx. p. quinquefasciatus* have developed resistance to insecticides in most regions of China. The neighboring downstream introns of the *vgsc* gene gradually decreased to one intron with the mutation of the *vgsc* gene. Mutations may originate from multiple mutation events rather than from a single origin, and populations lacking mutations may be genetically isolated.

**Graphical Abstract:**

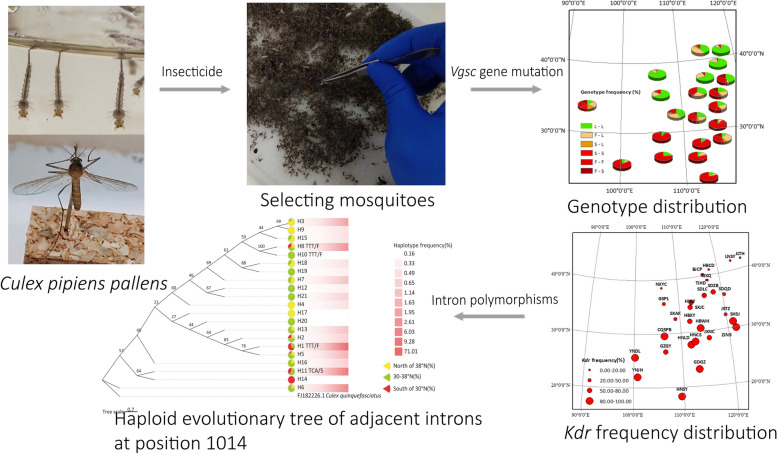

**Supplementary Information:**

The online version contains supplementary material available at 10.1186/s40249-024-01197-1.

## Background

*Culex pipiens pallens* and *Culex pipiens quinquefasciatus* are geographic subspecies of *Culex pipiens*, with *Cx. p. pallens* mainly distributed in northern China and *Cx. p. quinquefasciatus* dominating in southern China [1]. The larvae of *Cx. p. pallens* often breed in polluted water bodies, and the adult mosquitoes are the main domestic nuisance mosquito species in China in terms of biting and blood-sucking, and this species is an important target for urban mosquito control. These mosquitos can also transmit bancroftian filariasis [2].

Insecticides are widely used to control mosquito vectors because of their high efficiency, rapidity, broad spectrum and convenience. Since the first discovery of resistance in 1908 [3], resistance has been found in an increasing number of insect populations. One of the more widely used insecticides, pyrethroids act on sodium channels; therefore, sodium channel mutations result in resistance to insecticides, that is *kdr*. Research has shown that a mutation at site 1014 of the SII6 fragment of the para-type sodium channel causes resistance in mosquitoes [4–6]. Wild type L mutates to mutant type F [7]. Mutations at locus 1014 were found in both *Cx. p. pallens* and *Cx. p. quinquefasciatus* [7, 8]. The emergence of insecticide resistance in wild populations of mosquitoes is a complex process, which involves insecticide selection pressure during the evolutionary process from a wild homozygous (SS) to a wild/mutant heterozygote (RS), which then evolves into a mutant homozygous (RR) [9]. Mosquito resistance is caused by not only insecticide selection pressure but also individual migration, genetic drift, and neutral evolution during the long-term evolution of mosquitoes, which may affect the emergence and development of resistance [9]. Mutations at this locus induced by selective pressure from pyrethroid insecticides may lead to reduced nucleotide diversity in haplotypes [10, 11]. Variations in the neighboring intronic region of the gene can provide information about the long-term effects of pyrethroid insecticides on the evolution of mosquito populations [12]. Genetic diversity analysis of this locus, including the intronic region, can aid in understanding population genetic characteristics and predicting the risk of the localized emergence of resistance. However, at present, there are large time intervals and regional limitations in the monitoring of resistance in *Cx. p. pallens/Cx. p. quinquefasciatus*. For example, a survey of *Cx. p. quinquefasciatus* resistance in some areas of Anhui Province was performed in the 1990s, but the survey of *Cx. p. quinquefasciatus* resistance in north-central Anhui Province was assessed only after a period of 20 years [13]. To understand the current situation of *Cx. p. pallens/Cx. p. quinquefasciatus* in China, this study examined mutations in *Cx. p. pallens/Cx. p. quinquefasciatus* from 22 provincial-level administrative divisions. We analyzed the composition of downstream introns adjacent to the *vgsc* genes to understand the effects of pyrethroid insecticides on the long-term evolution of mosquito populations. This study provides insight into the impact of drug resistance on the long-term evolution of mosquitoes and provides a scientific basis for future directions of drug resistance monitoring.

## Methods

### Mosquito collection

Sampling was conducted from July to September 2021 in representative habitats in 22 provincial-level administrative divisions in China, including 28 populations (Table 1). The larval spoon method was used to collect larvae, and the mosquito trapping lamp method was used to collect adult mosquitoes [14]. A total of 8000 mosquitoes were collected from the 28 populations.

**Table 1:** *Culex pipiens pallens*/*Culex pipens quinquefasciatus* sample collection information

Codes	Location	Sampling amount	Acquisition time	Collection method	Latitude and longitude
North of 38°N					
JLTH	Tonghua, Jilin	90	Sep 2021	Light trap	(125°57′E, 41°40′N)
LNSY	Shenyang, Liaoning	150	Aug 2021	Light trap	(123°14′E, 41°29′N)
HBCD	Chengde, Hebei	100	Sep 2021	Larval spoon	(118°40′E, 40°51′N)
BJCP	Changping, Beijing	300	Sep 2021	Larval spoon	(116°18′E, 40°11′N)
TJHD	Hedong, Tianjin	100	Sep 2021	Light trap	(117°16′E, 39°9′N)
TJXQ	Xiqing, Tianjin	827	Sep 2021	Light trap	(117°4′E, 39°9′N)
NXYC	Yinchuan, Ningxia	100	Jul 2021	Light trap	(106°18′E, 38°21′N)
30°–38°N					
SDQD	Qingdao, Shandong	150	Sep 2021	Larval spoon	(120°31′E, 36°53′N)
SDZB	Zibo, Shandong	114	Sep 2021	Light trap	(118°3′E, 36°48′N)
SDLC	Liaocheng, Shandong	363	Sep 2021	Light trap	(115°59′E, 36°4′N)
GSPL	Pingliang, Gansu	389	Sep 2021	Light trap	(106°40′E, 35°32′N)
SXJC	Jincheng, Shanxi	529	Sep 2021	Light trap	(112°51′E, 35°30′N)
HNLY	Luoyang, Henan	300	Sep 2021	Light trap	(112°28′E, 34°22′N)
SXAK	Ankang, Shaanxi	170	Aug 2021	Larval spoon	(109°0′E, 32°42′N)
HBXY	Xiangyang, Hubei	100	Sep 2021	Light trap	(120°21′E, 32°18′N)
JSTZ	Taizhou, Jiangsu	500	Jun 2021	Larval spoon	(120°4′E, 32°10′N)
SHSJ	Songjiang, Shanghai	200	Aug 2021	Light trap	(121°18′E, 31°1′N)
HBWH	Wuhan, Hubei	130	Sep 2021	Light trap	(114°24′E, 30°38′N)
South of 30°N					
CQSPB	Shapingba, Chongqing	350	Sep 2021	Larval spoon	(106°23′E, 29°37′N)
ZJNB	Ningbo, Zhejiang	200	Sep 2021	Light trap	(121°27′E, 29°18′N)
JXNC	Nanchang, Jiangxi	120	Sep 2021	Light trap	(115°56′E, 28°33′N)
HNCS	Changsha, Hunan	351	Sep 2021	Light trap	(113°1′E, 28°16′N)
HNLD	Loudi, Hunan	72	Sep 2021	Light trap	(112°0′E, 27°43′N)
GZGY	Guiyang, Guizhou	210	Aug 2021	Light trap	(106°45′E, 26°33′N)
YNDL	Dali, Yunnan	200	Sep 2021	Light trap	(100°16′E, 25°36′N)
GDGZ	Guangzhou, Guangdong	322	Sep 2021	Light trap	(113°16′E, 23°13′N)
YNJH	Jinghong, Yunnan	400	Jan 2021	Larval spoon	(100°47′E, 22°1′N)
HNSY	Sanya, Hainan	300	Sep 2021	Larval spoon	(109°34′E, 18°16′N)

### Mosquito species identification

Morphological and molecular biology data were combined for mosquito species identification. Morphological identification was performed first. The ventral and inner leaves of the lateral plate of the male stem of *Cx. p. pallens/Cx. p. quinquefasciatus* are simple, with a wide leaf-like extension and a blunt end [2]. Subsequently, cytochrome C oxidase I (*COI*) in the mitochondrial DNA was selected for molecular biology classification and identification. The 656 bp *COI* gene was amplified with the following primers: COI-F: 5’-GGC CAA CAA ATC ATA AAG ATA TTG G-3’, COI-R: 5’-TAA ACT TCA GGG TGA CCA AAA AAT CA-3’ [15]. Each PCR mixture contained 12.5 μl of 2 × Easy*Taq*PCR SuperMix, 1 μl of each primer (10 μmol/L), 2 μl of template DNA, and ddH_2_O to a final volume of 25 μl. The thermocycler settings were as follows: 94 °C for 6 min; 35 cycles of 94 °C for 60 s, 51 °C for 60 s, and 72 °C for 60 s; and 72 °C for 8 min.

### *vgsc* gene amplification

The DNA of a single adult *Culex* mosquito was extracted using a magnetic bead-based microtissue genomic DNA extraction kit (Bioteke Corporation, Wuxi, China). The whole-genome DNA was used as a template to amplify the structural domain IIS6 of the *vgsc* gene using the following primers for CD: CD1-F: 5’-GAC CTG CCA CGG TGG AAC T-3’, CD2-R: 5’-TTG GAC AAA AGC AAG GCT AAG-3’ [9]. The PCR mixture contained 2 μl of whole-genome DNA, 12.5 μl of 2 × EasyTaqPCR SuperMix (TransGen Biotech, Beijing, China), 1 μl of each of the 10 μmol/L forward and reverse primers, and ddH_2_O to a final volume of 25 μl. The PCR conditions were 94 ℃ for 3 min; 35 cycles of 94 ℃ for 30 s, 60 ℃ for 30 s, and 72 ℃ for 30 s; and 72 ℃ for 8 min. The samples were stored at 4 ℃. PCR amplification products were detected using 1% agarose gel electrophoresis, and samples with clear bands and no trailing were selected for forward sequencing.

### Data analysis

Seqman [16] software was used to compare and analyze the peak maps of the *vgsc* gene, determine the allele and genotype of the test insect, and calculate *kdr* frequency. MUSCLE comparison [17] of sequences was performed using MEGA 11 [18]. MEGA 11 was used to construct neighbor-joining (NJ) tree. GeneDoc [19] was used to plot sequence comparisons. The TCS network [20] was constructed using PopART [21], and the resulting haplotype network was plotted. *kdr* frequency was calculated as follows: $kdr\;\mathrm{frequency}=\frac{RR+\frac{RS}2}N$ where RR is resistant pure or resistant homozygote, and RS is a heterozygote genotype of wildtype and resistance genes.

## Results

### Polymorphisms at the 1014 locus of the *vgsc* gene

A total of 1308 sequences of domain II of the *vgsc* gene were obtained in this study. Codon 1014 of structural domain II was analyzed, and six alleles and six genotypes were identified (Table 2). The first category of mutations was nonsynonymous mutations. Nonsynonymous mutations were categorized into two types: the mutation of leucine to phenylalanine (L1014F) and the mutation of leucine to serine (L1014S). The second category was the base double mutation of L1014F/L1014S, where leucine is mutated to both phenylalanine and serine at the same time (F1014S). The third category was the base synonymous mutation (L1014L). The six alleles obtained were TTA/L and CTA/L, encoding leucine; TTT/F and TTC/F, encoding phenylalanine; and TCT/S and TCA/S, encoding serine. The frequencies of TTT/F and TTA/L were greater, with frequencies of 47.17% and 43.58%, respectively. The genotypes included wild-type homozygous L/L, wild-type/mutant heterozygous F/L and S/L, mutant homozygous F/F and S/S, and mutant heterozygous F/S, with frequencies ranging from 37.77% (F/F) to 1.61% (S/L).

**Table 2:** Detection results of target sites in the *Cx. pipiens pallens*/*Cx. pipiens quinquefasciatus vgsc* gene

Type of mutation at 1014 locus (*n* = 1308)	*n* (%)
Allelic genotype	
TTA/L	1141 (43.58)
CTA/L	2 (0.08)
TTT/F	1235 (47.17)
TTC/F	19 (0.73)
TCA/S	219 (8.37)
TCT/S	2 (0.08)
Genotype	
L/L	461 (35.24)
F/L	198 (15.14)
S/L	21 (1.61)
F/F	494 (37.77)
S/S	65 (4.97)
F/S	69 (5.28)

TTA, CTA, TTT, TTC, TCA, and TCT are codons. L, F, and S are concatenated bases representing leucine, phenylalanine, and serine, respectively. TTA and CTA encode leucine. TTT and TTC encode phenylalanine. TCA and TCT encode serine. *n* represents the total number of test mosquitos

### Spatial distribution of the genes

The TTT/F mutant allele was detected in all 28 populations (Table 3). The frequency of TTT/F ranged from 98.94% (YNJH) to 2.08% (JLTH). TCT/S was detected in the JXNC and SDLC populations at a frequency of 1.20%. CTA/L was found only in the SDLC population at a frequency of 2.38%. The F/S mutation was detected in 14 populations of ZJNB, HNSY, and SDZB, with frequencies ranging from 26.67% (ZJNB) to 1.19% (HNLY). The frequency of S/L was less than 10.00% in nine populations in which it was detected. In addition, F/F was detected in all 28 populations, with frequencies ranging from 97.87% (YNJH) to 2.08% (JLTH). Overall, TTT/F was the predominant mutant allele type (Table 4).

**Table 3:** Allele distribution at locus 1014 in *Cx. pipiens pallens/Cx. pipiens quinquefasciatus* at different latitudes

Codes	Number	TTA/L, *n* (%)	CTA/L, *n* (%)	TTT/F, *n* (%)	TTC/F, *n* (%)	TCA/S, *n* (%)	TCT/S, *n* (%)
North of 38° N							
JLTH	48	94 (97.92)	0 (0.00)	2 (2.08)	0 (0.00)	0 (0.00)	0 (0.00)
LNSY	42	75 (89.28)	0 (0.00)	7 (8.33)	2 (2.38)	0 (0.00)	0 (0.00)
HBCD	41	73 (89.02)	0 (0.00)	9 (10.98)	0 (0.00)	0 (0.00)	0 (0.00)
BJCP	89	113 (63.48)	0 (0.00)	58 (32.58)	1 (0.56)	6 (3.37)	0 (0.00)
TJHD	47	76 (80.85)	0 (0.00)	18 (19.15)	0 (0.00)	0 (0.00)	0 (0.00)
TJXQ	47	82 (87.23)	0 (0.00)	12 (12.77)	0 (0.00)	0 (0.00)	0 (0.00)
NXYC	40	72 (90.00)	0 (0.00)	7 (8.75)	0 (0.00)	1 (1.25)	0 (0.00)
30°–38° N							
SDQD	47	72 (76.60)	0 (0.00)	20 (21.28)	1 (1.06)	1 (1.06)	0 (0.00)
SDZB	45	40 (44.44)	0 (0.00)	36 (40.00)	0 (0.00)	14 (15.56)	0 (0.00)
SDLC	42	25 (29.76)	2 (2.38)	42 (50.00)	0 (0.00)	14 (16.67)	1 (1.19)
GSPL	34	51 (75.00)	0 (0.00)	12 (17.65)	1 (1.47)	4 (5.88)	0 (0.00)
SXJC	42	40 (47.62)	0 (0.00)	39 (46.43)	1 (1.19)	4 (4.76)	0 (0.00)
HNLY	84	50 (29.76)	0 (0.00)	90 (53.57)	0 (0.00)	28 (16.67)	0 (0.00)
SXAK	46	53 (57.61)	0 (0.00)	35 (38.04)	0 (0.00)	4 (4.35)	0 (0.00)
HBXY	39	30 (38.46)	0 (0.00)	33 (42.31)	0 (0.00)	15 (19.23)	0 (0.00)
JSTZ	48	65 (67.71)	0 (0.00)	21 (21.88)	3 (3.13)	7 (7.29)	0 (0.00)
SHSJ	38	10 (13.16)	0 (0.00)	32 (42.11)	8 (10.53)	26 (34.21)	0 (0.00)
HBWH	37	5 (6.76)	0 (0.00)	54 (72.97)	2 (2.70)	13 (17.57)	0 (0.00)
South of 30° N							
CQSPB	42	8 (9.52)	0 (0.00)	69 (82.14)	0 (0.00)	7 (8.33)	0 (0.00)
ZJNB	45	14 (15.56)	0 (0.00)	60 (66.67)	0 (0.00)	16 (17.78)	0 (0.00)
JXNC	41	23 (28.05)	0 (0.00)	44 (53.66)	0 (0.00)	14 (17.07)	1 (1.22)
HNCS	40	9 (11.25)	0 (0.00)	69 (86.25)	0 (0.00)	2 (2.5)	0 (0.00)
HNLD	39	11 (14.10)	0 (0.00)	55 (70.51)	0 (0.00)	12 (15.38)	0 (0.00)
GZGY	22	10 (22.73)	0 (0.00)	34 (77.27)	0 (0.00)	0 (0.00)	0 (0.00)
YNDL	90	24 (13.33)	0 (0.00)	156 (86.67)	0 (0.00)	0 (0.00)	0 (0.00)
GDGZ	41	13 (15.85)	0 (0.00)	69 (84.15)	0 (0.00)	0 (0.00)	0 (0.00)
YNJH	47	1 (1.06)	0 (0.00)	93 (98.94)	0 (0.00)	0 (0.00)	0 (0.00)
HNSY	45	0 (0.00)	0 (0.00)	59 (65.56)	0 (0.00)	31 (34.44)	0 (0.00)

TTA, CTA, TTT, TTC, TCA, and TCT are codons. L, F, and S are concatenated bases representing leucine, phenylalanine, and serine, respectively. TTA and CTA encode leucine. TTT and TTC encode phenylalanine. TCA and TCT encode serine. *n* represents the total number of test mosquitos

**Table 4:** Genotype distribution at locus 1014 in *Cx. pipiens pallens/Cx. pipiens quinquefasciatus* at different latitudes

Codes	Number	L/L, *n* (%)	F/L, *n* (%)	F/F, *n* (%)	S/L, *n* (%)	S/S, *n* (%)	F/S, *n* (%)
North of 38° N							
JLTH	48	47 (97.92)	0 (0.00)	1 (2.08)	0 (0.00)	0 (0.00)	0 (0.00)
LNSY	42	35 (83.33)	5 (11.90)	2 (4.76)	0 (0.00)	0 (28.34)	0 (0.00)
HBCD	41	35 (85.37)	3 (7.32)	3 (7.32)	0 (0.00)	0 (17.85)	0 (0.00)
BJCP	89	35 (39.33)	39 (43.82)	10 (11.24)	4 (4.49)	1 (49.24)	0 (0.00)
TJHD	47	33 (70.21)	10 (21.28)	4 (8.51)	0 (0.00)	0 (45.27)	0 (0.00)
TJXQ	47	37 (78.72)	8 (17.02)	2 (4.26)	0 (0.00)	0 (36.22)	0 (0.00)
NXYC	40	33 (82.50)	5 (12.50)	1 (2.50)	1 (2.50)	0 (31.25)	0 (0.00)
30°–38° N							
SDQD	47	30 (63.83)	12 (25.53)	4 (8.51)	0 (0.00)	0 (54.32)	1 (2.13)
SDZB	45	19 (42.22)	2 (4.44)	12 (26.67)	0 (0.00)	2 (9.88)	10 (22.22)
SDLC	42	9 (21.43)	8 (19.05)	14 (33.33)	1 (2.38)	3 (45.35)	7 (16.67)
GSPL	34	23 (67.65)	5 (14.71)	4 (11.76)	0 (0.00)	2 (43.25)	0 (0.00)
SXJC	42	14 (33.33)	12 (28.57)	13 (30.95)	0 (0.00)	1 (68.03)	2 (4.76)
HNLY	84	16 (19.05)	17 (20.24)	36 (42.86)	1 (1.19)	13 (24.09)	1 (1.19)
SXAK	46	18 (39.13)	15 (32.61)	10 (21.74)	2 (4.35)	1 (70.89)	0 (0.00)
HBXY	39	10 (25.64)	7 (17.95)	11 (28.21)	3 (7.69)	4 (46.02)	4 (10.26)
JSTZ	48	28 (58.33)	5 (10.42)	9 (18.75)	4 (8.33)	1 (21.7)	1 (2.08)
SHSJ	38	3 (7.89)	2 (5.26)	18 (47.37)	2 (5.26)	11 (13.85)	2 (5.26)
HBWH	37	1 (2.70)	3 (8.11)	24 (64.86)	0 (0.00)	4 (21.91)	5 (13.51)
South of 30° N							
CQSPB	42	4 (9.52)	0 (0.00)	33 (78.57)	0 (0.00)	2 (0.00)	3 (7.14)
ZJNB	45	1 (2.22)	12 (26.67)	18 (40.00)	0 (0.00)	2 (59.26)	12 (26.67)
JXNC	41	4 (9.76)	12 (29.27)	12 (29.27)	3 (7.32)	2 (71.39)	8 (19.51)
HNCS	40	4 (10.00)	1 (2.50)	33 (82.50)	0 (0.00)	0 (6.25)	2 (5.00)
HNLD	39	3 (7.69)	5 (12.82)	25 (64.10)	0 (0.00)	6 (32.87)	0 (0.00)
GZGY	22	5 (22.73)	0 (0.00)	17 (77.27)	0 (0.00)	0 (0.00)	0 (0.00)
YNDL	90	9 (10.00)	6 (6.67)	75 (83.33)	0 (0.00)	0 (7.41)	0 (0.00)
GDGZ	41	5 (12.20)	3 (7.32)	33 (80.49)	0 (0.00)	0 (17.85)	0 (0.00)
YNJH	47	0 (0.00)	1 (2.13)	46 (97.87)	0 (0.00)	0 (4.53)	0 (0.00)
HNSY	45	0 (0.00)	0 (0.00)	24 (53.33)	0 (0.00)	10 (0.00)	11 (24.44)

L, F, and S are concatenated bases representing leucine, phenylalanine, and serine, respectively

The allelic and genotypic compositions of geographic populations within the same latitudinal range were similar. *kdr* frequency increased with decreasing latitude. The geographic populations north of 38° N predominantly contained TTA/L and L/L, with the allelic mutation being TTT/F, and they had fewer homozygous mutations and a single mutation type. The frequency of mutant alleles increased in populations at 30°–38° N latitude compared to high-latitude populations, with the emergence of TCA/S and TCT/S mutant allele types and the mutant heterozygote F/S. Alleles and genotypes tended to be enriched in mid-latitude populations. The percentage of mutant alleles in some populations south of 30° N latitude was more than 90.00%, and these populations were dominated by TTT/F. Lower-latitude populations exhibited increased mutant purities, mainly F/F, and the alleles and genotypes were more homogeneous. Geographic populations with frequencies less than 20.00% were concentrated north of 38° N. Frequencies of 20.00%–80.00% were mostly found at 30°–38° N. Geographic populations south of 30° N latitude had frequencies greater than 80.00%. Within the same latitudinal range, the frequency was significantly greater in the large city populations than in the other populations. The frequencies of BJCP, HBWH, SHSJ, CQSPB, and GDGZ were 36.52%, 93.24%, 86.84%, 90.48%, and 84.15%, respectively, which were greater than those of other populations at the same latitude (Table 5).

**Table 5:** *kdr* frequency of *Cx. pipiens pallens*/ *Cx. pipiens quinquefasciarus* in 28 geographical populations

Codes	Number	SS, *n* (%)	RS, *n* (%)	RR_1_, *n* (%)	RR_2_, *n* (%)	*kdr* frequency, %
North of 38°N						
JLTH	48	47 (97.92)	0 (0.00)	1 (2.08)	0 (0.00)	2.08
LNSY	42	35 (83.33)	5 (11.91)	2 (4.76)	0 (0.00)	10.71
HBCD	41	35 (85.37)	3 (7.32)	3 (7.32)	0 (0.00)	10.98
BJCP	89	35 (39.33)	43 (48.31)	11 (12.36)	0 (0.00)	36.52
TJHD	47	33 (70.21)	10 (21.28)	4 (8.51)	0 (0.00)	19.15
TJXQ	47	37 (78.72)	8 (17.02)	2 (4.26)	0 (0.00)	12.77
NXYC	40	33 (82.50)	6 (15.00)	1 (2.50)	0 (0.00)	10.00
30°–38°N						
SDQD	47	30 (63.83)	12 (25.53)	4 (8.51)	1 (2.13)	23.40
SDZB	45	19 (42.22)	2 (4.44)	14 (31.11)	10 (22.22)	55.56
SDLC	42	9 (21.42)	9 (21.43)	17 (40.48)	7 (16.67)	67.86
GSPL	34	23 (67.65)	5 (14.71)	6 (17.65)	0 (0.00)	25.00
SXJC	42	14 (33.34)	12 (28.57)	14 (33.33)	2 (4.76)	52.38
HNLY	84	16 (19.05)	18 (21.43)	49 (58.33)	1 (1.19)	70.24
SXAK	46	18 (39.13)	17 (36.96)	11 (23.91)	0 (0.00)	42.39
HBXY	39	10 (25.64)	10 (25.64)	15 (38.46)	4 (10.26)	61.54
JSTZ	48	28 (58.33)	10 (20.83)	9 (18.75)	1 (2.08)	32.29
SHSJ	38	3 (7.89)	4 (10.53)	29 (76.32)	2 (5.26)	86.84
HBWH	37	1 (2.70)	3 (8.11)	28 (75.68)	5 (13.51)	93.24
South of 30°N						
CQSPB	42	4 (9.52)	0 (0.00)	35 (83.33)	3 (7.14)	90.48
ZJNB	45	1 (2.22)	12 (26.67)	20 (44.44)	12 (26.67)	84.44
JXNC	41	4 (9.76)	15 (36.59)	14 (34.15)	8 (19.51)	71.95
HNCS	40	4 (10.00)	33 (82.50)	1 (2.50)	2 (5.00)	88.75
HNLD	39	3 (7.69)	5 (12.82)	31 (79.49)	0 (0.00)	85.90
GZGY	22	5 (22.73)	0 (0.00)	17 (77.27)	0 (0.00)	77.27
YNDL	90	9 (10.00)	6 (6.67)	75 (83.33)	0 (0.00)	86.67
GDGZ	41	5 (12.20)	3 (7.32)	33 (80.49)	0 (0.00)	84.15
YNJH	47	0 (0.00)	1 (2.13)	46 (97.87)	0 (0.00)	98.94
HNSY	45	0 (0.00)	0 (0.00)	34 (75.56)	11 (24.44)	100.00
Laboratory strain						
BJ	94	66 (70.21)	26 (27.66)	2 (2.13)	0 (0.00)	15.96

SS is a sensitive gene homozygous. RR_1_ is a resistance gene homozygous (F/F and S/S). RR_2_ is a resistance gene heterozygote (F/S). RS is a heterozygote for the sensitive gene and the resistance gene

### Characterization of neighboring downstream introns

Twenty-one haplotypes were obtained from 614 D2 sequences. Sequence analysis revealed that the mutant gene of *Cx. p. pallens/Cx. p. quinquefasciatus* was adjacent to a downstream intron in which the 5' end was GT, and the 3' end was AG (see Additional file 1). Overall, the total AT content was greater than the total GC content. The difference between AT and GC was most pronounced at the first base site, and A was predominant at all but the second base site (Table 6). These findings suggest that AT values increase in the downstream intron adjacent to the mutant gene of *Cx. p. pallens/Cx. p. quinquefasciatus*, and adenine (A) is preferred at the codon site.

**Table 6:** Sequence characteristics of downstream introns adjacent to the mutant gene

Base site	A, %	T, %	C, %	G, %
First base site (108 bp^a^)	30.2	28.8	23.8	17.2
Second base site (110 bp^a^)	26.7	28.4	22.8	22.2
Third base site (109 bp^a^)	29.9	25.8	23.9	20.4
Total fragment (326 bp^a^)	28.9	27.7	23.5	19.9

T represents thymine. A represents adenine. G represents guanine. C represents cytosine. % Indicates the proportion of the nucleic acid in the fragment. ^a^represents the fragment length (bp), which is the average fragment length of 28 geographical populations

### Haplotype analysis of neighboring downstream introns to the mutant gene

**Fig. 1:**
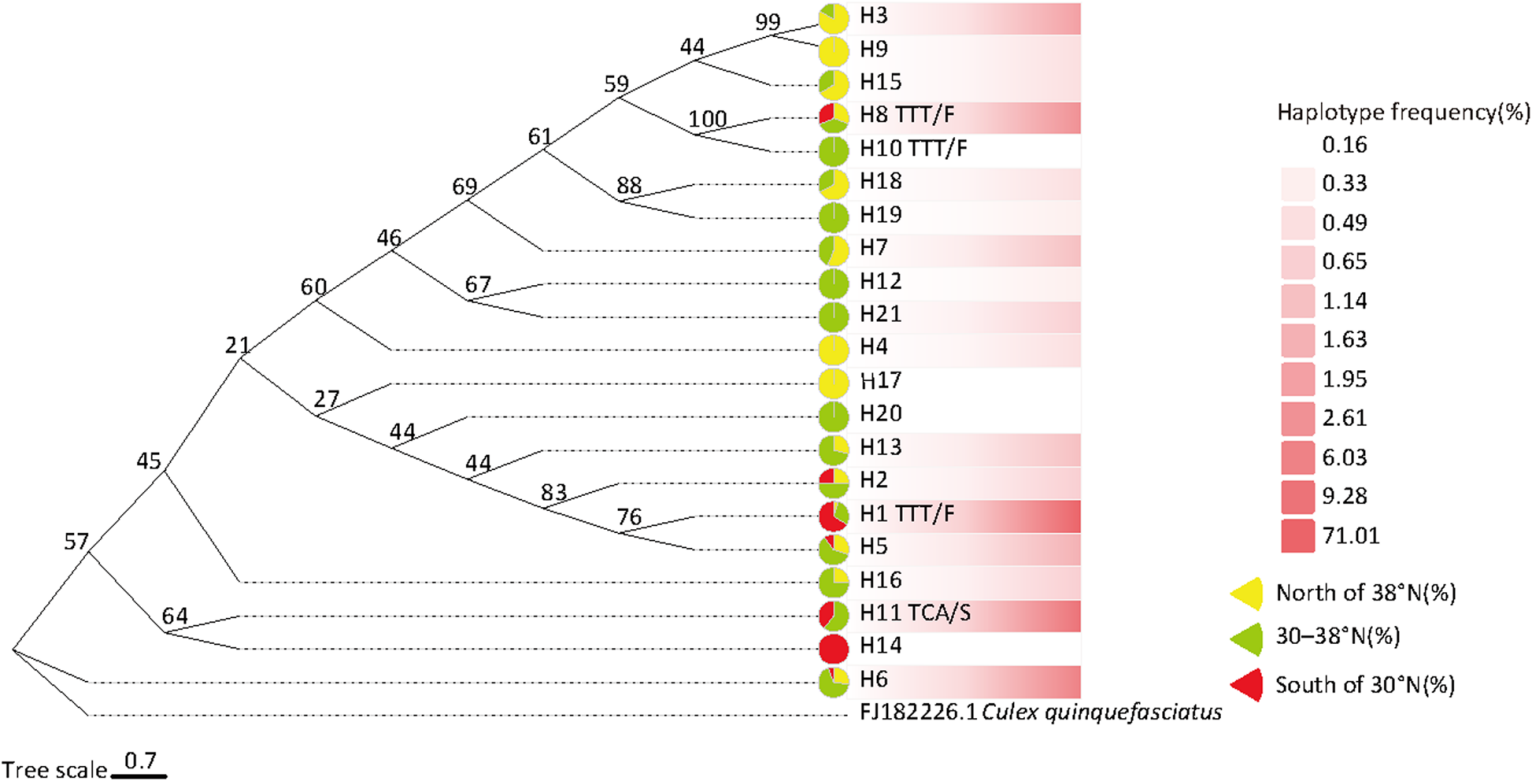
Haploid evolutionary tree of adjacent introns at position 1014 of the *vgsc* gene. Note: All unlabeled data are TTA/L. Pie charts indicate percentages at different latitudes. FJ182226.1 from NCBI, is the *vgsc* gene of *Culex quinquefasciatus*

**Fig. 2:**
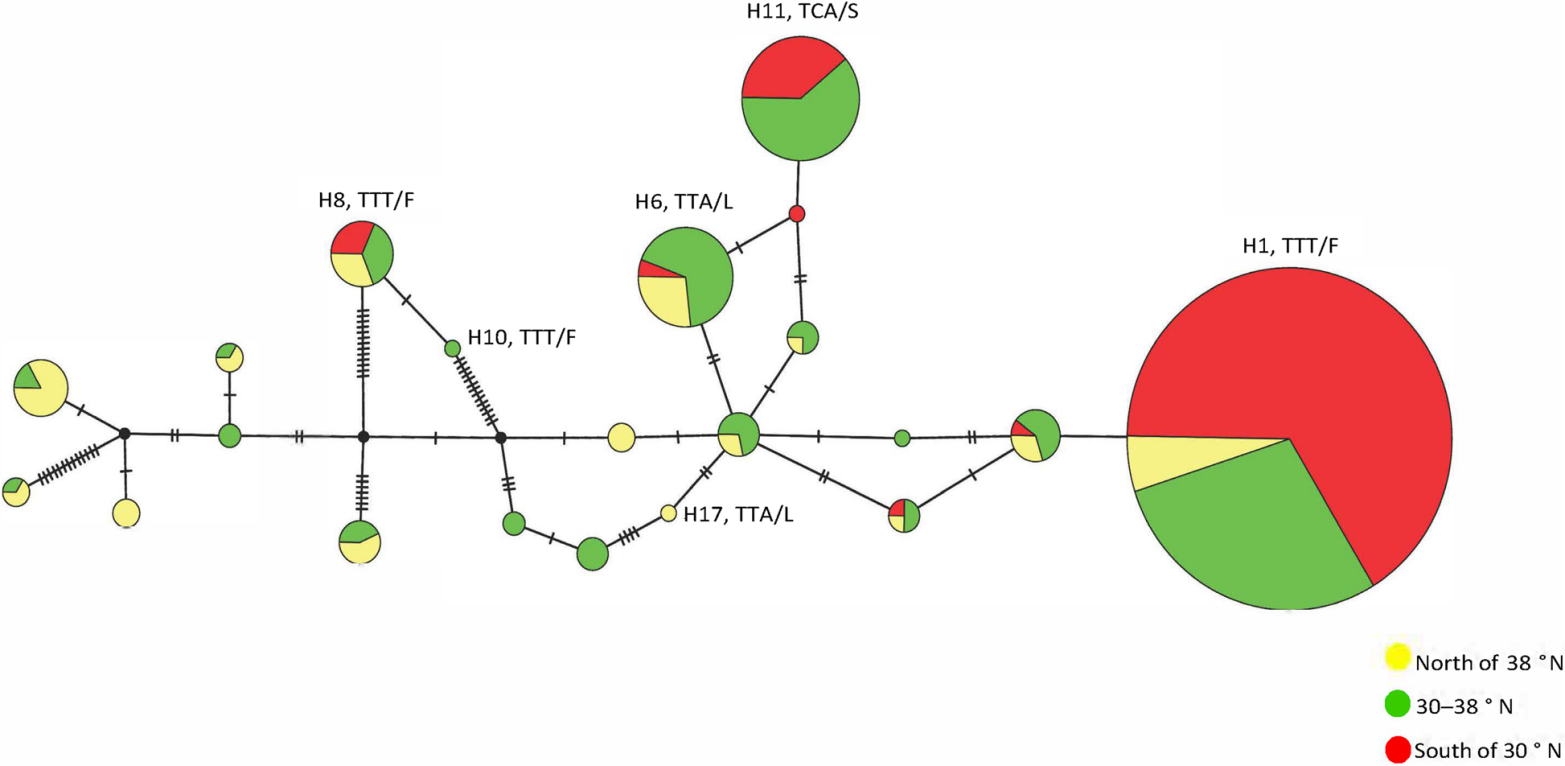
Haploid network of adjacent introns at position 1014 of the *vgsc* gene. Note: The colored circles indicate the detected haplotypes. The black dots indicate deletions or haplotypes not actually observed in the sample. The circle size indicates the haplotype frequency. Consecutive vertical lines indicate the presence of mutations between haplotypes. The colored dashed lines indicate different clusters. Unlabeled lines are TTA/L

The phylogenetic evolutionary tree of twenty-one intronic haplotypes showed that most of the intronic haplotypes were clustered in one large lineage, with H6 and H16 as independent branches. The intronic haplotype of TTA/L is widely distributed. Mutant allele-linked intronic haplotypes are distributed mainly within the same major lineage, in which the TTT/F intronic haplotypes H8 and H10 originate from the same branch and are sister groups to each other. The neighboring intronic haplogroups H1 and H5 of TTT/F and TTA/L are sister groups to each other. The neighboring intronic haplotypes H11 and H14 of TCA/S and TTA/L are sister groups to each other (Fig. 1). The haplotype network diagram shows that H17 is the dominant haplotype (Fig. 2). H17 may be the ancestral sequence from which the other haplotypes may have evolved. The mutant alleles TTT/F and TCA/S are derived from different TTA/Ls, with at least one sensitive haplotype on each branch. The mutant allele TCA/S has only one haplotype, H11, in the neighboring downstream intron, and TTT/F has three haplotypes, H1, H8, and H10, whereas the wild-type allele TTA/L has 17 haplotypes in the neighboring downstream intron (Fig. 2). It can be inferred that the neighboring downstream introns of the mutant allele are derived from the introns of the wild-type allele and are homologous to some of the wild-type introns. The introns of the mutant allele tend to stabilize, suggesting that the mutation is correlated with the introns.

## Discussion

A total of three mutations were detected in this study: one was a nonsynonymous mutation, in which leucine L1014 was mutated to phenylalanine L1014F (TTA-TTT) or serine L1014S (TTA-TCA). The second was a base double mutation of L1014F/L1014S, in which leucine L1014 is mutated to both phenylalanine and serine F1014S. The third was a base synonymous mutation of L1014L (TTA-CTA). Consistent with the results of existing studies [22–25], the L1014F mutation is not conserved. The existence of multiple mutation types at locus 1014 suggested that the mutations in *Cx. p. pallens/Cx. p. quinquefasciatus* in China tend to be diversified, and the genotype is polymorphic. Increased mutational orientation of locus 1014 under insecticide selective pressure and enhanced adaptation to the environment. The present study revealed significant geographic variation in the type, number and frequency of mutations. The allelic and genotypic composition of *Cx. p. pallens/Cx. p. quinquefasciatus* varies with latitude, from single to diverse, before shifting to single. The genotypes are more monotypic north of 38° N latitude. The genotypes in the 30–38° N region are diverse. Populations south of 30° N latitude are homogeneous in terms of allelic and genotypic composition. Populations north of 38° N had a high percentage of L/L, while populations south of 30° N had more mutant genotypes, with no wild pureblood genotypes detected in YNJH and HNSY and less than 5% of wild types in HBWH and ZJNB. Populations south of 30° N latitude had a high mutation frequency and a small percentage of sensitive genotypes. The adaptability of the mutant phenotypes of *Cx. p. pallens/Cx. p. quinquefasciatus* is increasing under the selective pressure of insecticides. Mutant genes can be stably inherited in populations, suggesting that genes may be conserved in the wild.

In this study, the synonymous mutation CTA/L was detected only in the Liaocheng population in Shandong, China. Most of the mutations at locus 1014 of *Aedes albopictus* from the Anhui and Hunan Zhuzhou populations were CTA/L. It is possible that CTA/L is nonmutated in *Aedes albopictus* because locus 1014 has a CTA/L background. In contrast, the wild-type *Cx. p. pallens/Cx. p. quinquefasciatus* is TTA/L, and although CTA and TTA both encode leucine, CTA may be a mutation in *Cx. p. pallens/Cx. p. quinquefasciatus*. In the Zibo population, the CTA and TTC allele genotypes were not detected, in contrast to the results of Wei's study [24]. This difference may be due to the following reasons. The studies used different sampling habitats and sampling times. Convenient transportation, frequent transportation of goods, and well-developed tourism increase the chances of mosquitoes' passive movement, which increases the possibility of gene exchange between populations and changes the frequency of allele genotypes of the populations. And the populations had a low content of mutant alleles.

Resistance has developed in *Cx. p. pallens/Cx. p. quinquefasciatus* in most areas. The laboratory strain of *Cx. p. pallens* (BJ strain) had a resistance frequency of 15.96% and a mortality rate of 50.00% under exposure to a diagnostic dose of deltamethrin of 0.025%. The BJ strain was resistant to deltamethrin [26]. Combining the BJ bioassay results and the frequency, it can be seen that field populations with a frequency < 20.00% have developed resistance to pyrethroid insecticides. The greater the frequency is, the lower the sensitivity to pyrethroid insecticides. This indicates that the light-colored *Culex* mosquitoes in most parts of the country have become resistant to the insecticide. Even populations with low frequencies may have developed resistance. When the frequency of *vgsc* alleles in *Culex pipiens* is low, cytochrome P450-mediated metabolic resistance dominates. With continued selection pressure, as the number of screening generations increases, the expression of P450 genes tends to stabilize, genetic differences between individuals in the population decrease, the actual heritability of drug resistance in the population increases, and *vgsc* mutations dominate. A combination of biological and molecular methods should be used to determine the level of resistance in populations, especially those with low frequencies, in a comprehensive manner.

Wild population resistance is regional in nature. The frequency increases as the latitude decreases. This study revealed that the mutation in *Cx. p. pallens/Cx. p. quinquefasciatus* in China exhibits significant spatial heterogeneity. The number and frequency of mutations showed significant geographic differences. Geographic populations with frequencies < 20.00% were mainly concentrated north of 38° N latitude. However, geographic populations south of 30° N latitude have frequencies > 80.00%. The populations south of 30° N latitude, such as the HNSY, YNJH, CQSPB and HBWH populations, had frequencies > 90.00%. No wild-type allele genotypes were detected in the HNSY population, with a mutation frequency of 100.00%. This could be because temperatures are higher at lower latitudes, and temperature is one of the most important factors affecting the growth cycle of mosquitoes and the level of breeding. Tire-causing *Cx. p. quinquefasciatus* were caught throughout the year in Hainan and Guangdong. In Jilin and Liaoning, the peak occurred in August, and after October, there were basically no catches of *Cx. p. pallens* [27]. Mosquitoes overwinter differently, affecting the number of mosquitoes in the second year, which in turn affects the genetics of resistance. The more mosquitoes that overwinter, the more mosquitoes there will be in the following year. The greater the percentage of resistant mosquitoes that survive overwintering is, the greater the probability of resistance inheritance. Shandong's *Cx. p. pallens* overwinter at high temperatures and high humidity and in light-sheltered kilns, and the larvae cannot overwinter [28]. *Cx. p. quinquefasciatus* in Jiangsu overwinters in underground garages, stairwells on the ground floor of buildings, and private houses on the outskirts of the city in different forms [29]. On the other hand, Yunnan Province has a high incidence of dengue fever [30], and Chongqing had a dengue outbreak in 2019 [31]. Therefore, the frequency, dose and duration of insecticide use are greater in these areas than in others. Insecticide residue levels are increased at mosquito breeding sites. The duration of mosquito exposure to insecticides increases, leading to increased mosquito resistance. The higher *kdr* frequency also converges across urban areas. The frequency of pesticide use is related not only to natural factors such as latitude and temperature but also to social factors such as economic, demographic. BJCP, SHSJ, and HBWH are large cities in terms of population, economy, and transportation. These cities are densely populated and serve as important transportation hubs with high logistics and people flow. Developed regions have a long history of insecticide use, and mosquitoes are exposed to insecticides for long periods of time, increasing cumulative toxicity. The frequency of insecticide use is greater in large cities within the same latitude. Mosquitoes are more likely to develop insecticide resistance.

This study only included mosquitoes collected in 2021 and lacked previous data. In recent years, due to its relationship with dengue fever, research on *Aedes albopictus* has been increasing, and research on *Cx. p. pallens* has correspondingly decreased. The main focus of this study was to investigate the haplotypes of adjacent introns downstream of locus 1014 using the appropriate sequences. The detected *vgsc* genes may not necessarily contain the complete introns required for research, making it impossible to obtain additional historical sequence data for *Cx. p. pallens*. Another approach is to compare functional genes (such as *vgsc* genes) with neutral markers (such as mitochondrial DNA), which may more accurately demonstrate the combined impact of demographic and selection factors on population structure.

## Conclusions

This study described the spatial distribution of mutations in 28 populations of *Cx. p. pallens/Cx. p. quinquefasciatus* in China. The mosquitoes in most areas had developed insecticide resistance, and the degree of resistance was regional. The adjacent downstream intron of the gene was polymorphic and associated with the mutation. These results highlight the importance of monitoring the level of pyrethroid resistance in field mosquitoes, and limiting the spread of alleles will help to prevent the development of insecticide resistance. Establishing a long-term monitoring mechanism for insecticide resistance and understanding the development trend of insecticide resistance are helpful for implementing integrated mosquito control measures according to local conditions.

### Supplementary Information

**Supplementary Material 1.**

**Supplementary Material 2.**

## Data Availability

The datasets used and analyzed during the current study are available from the corresponding author upon reasonable request.

## Abbreviations

*vgsc*: Voltage-gated sodium channel
*kdr*: Knockdown resistance
NJ: Neighbor-joining
SS: Wild homozygous
RS: Wild/mutant heterozygote
RR: Mutant homozygous
*COI*: Cytochrome C oxidase I
