# Supplementary material for: Mutations and intron polymorphisms in voltage-gated sodium channel genes of different geographic populations of Culex pipiens pallens/Culex pipiens quinquefasciatus in China
Source: Infect Dis Poverty. 2024 Apr 15;13:29. doi: 10.1186/s40249-024-01197-1 (PMC11017551; doi:10.1186/s40249-024-01197-1)
Supplement: Supplementary file 1 — Supplementary Material 1. [file 40249_2024_1197_MOESM1_ESM.docx]

Additional file 1: Sequence characteristics


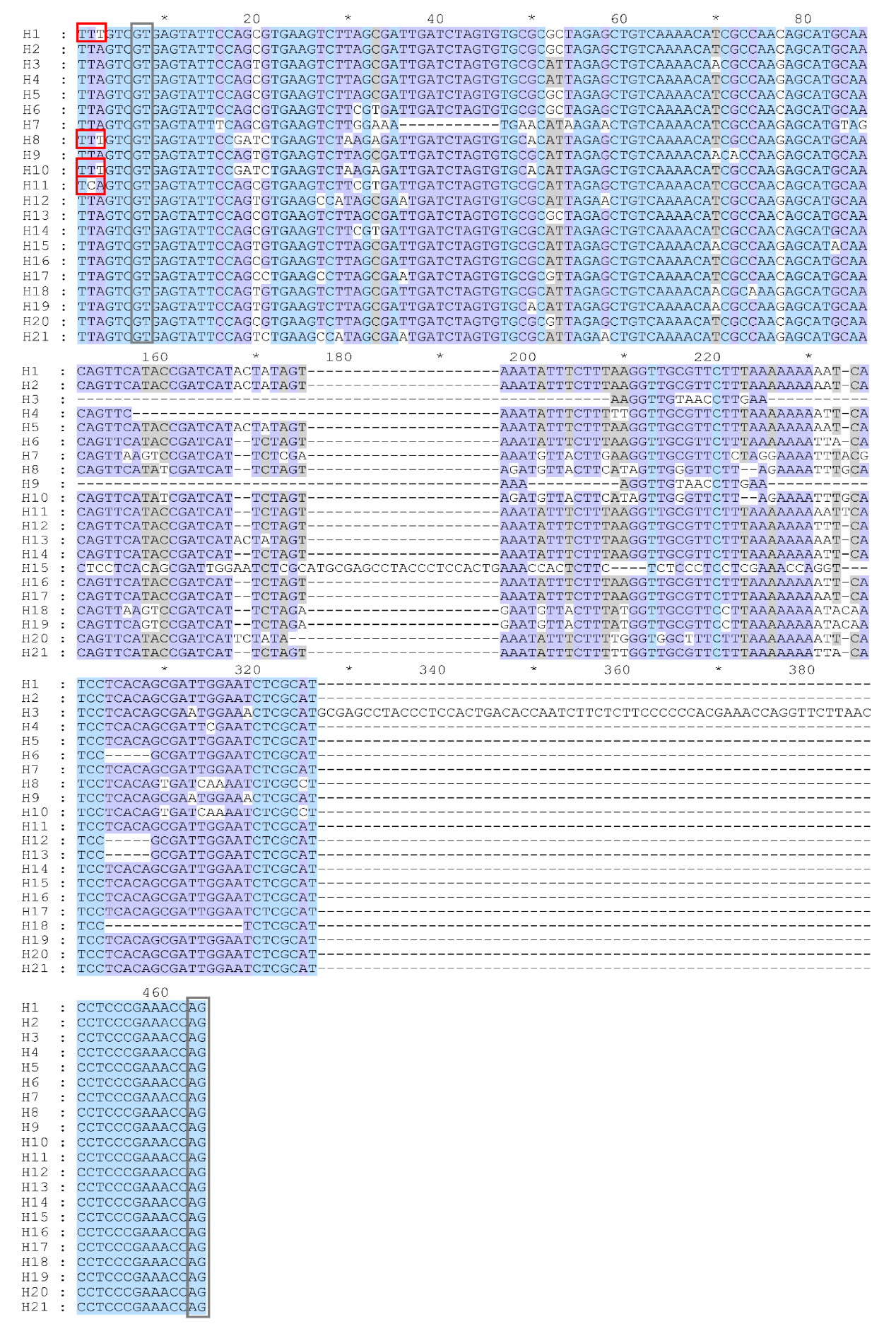


Note: H1-H21 indicate haplotype numbers. Red boxes indicate gene 1014 locus mutation allele genotypes. The black boxes represent the 5 'and 3' ends of introns, respectively. Blue color indicates identical codons. - indicates vacancies
