# Supplementary material for: Mutations and intron polymorphisms in voltage-gated sodium channel genes of different geographic populations of Culex pipiens pallens/Culex pipiens quinquefasciatus in China
Source: Infect Dis Poverty. 2024 Apr 15;13:29. doi: 10.1186/s40249-024-01197-1 (PMC11017551; doi:10.1186/s40249-024-01197-1)
Supplement: Supplementary file 2 — Supplementary Material 2. [file 40249_2024_1197_MOESM2_ESM.docx]

Additional file 2: Gel electrophoresis of PCR amplification products at *vgsc* gene


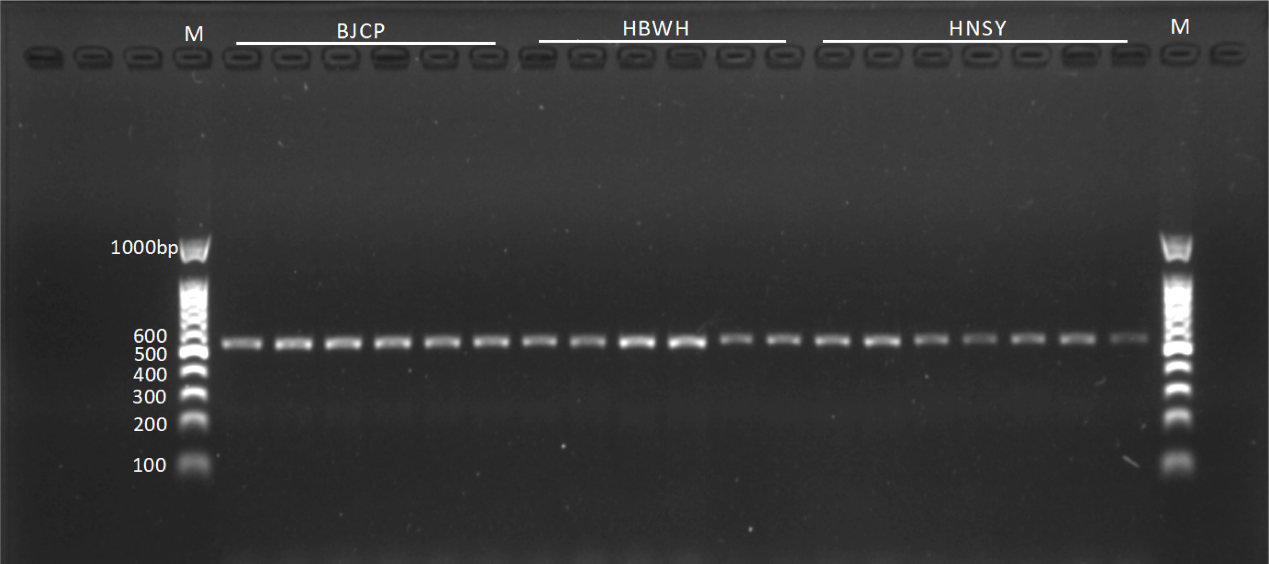


Note: M is the abbreviation for marker.
